# Amino acids, post-translational modifications, nitric oxide, and oxidative stress in serum and urine of long COVID and ex COVID human subjects

**DOI:** 10.1007/s00726-023-03305-1

**Published:** 2023-07-29

**Authors:** Marie Mikuteit, Svetlana Baskal, Sandra Klawitter, Alexandra Dopfer-Jablonka, Georg M. N. Behrens, Frank Müller, Dominik Schröder, Frank Klawonn, Sandra Steffens, Dimitrios Tsikas

**Affiliations:** 1 Department of Rheumatology and Immunology, Hannover Medical School, Hannover, Germany; 2 Hannover Medical School, Dean’s Office–Curriculum Development, Hannover, Germany; 3 Hannover Medical School, Institute of Toxicology, Core Unit Proteomics, Carl-Neuberg-Strasse 1, 30625 Hannover, Germany; 4 Institute for Information Engineering, Ostfalia University of Applied Sciences, Wolfenbüttel, Germany; 5 German Center for Infection Research (DZIF), Partner Site Hannover-Braunschweig, Hannover, Germany; 6 Centre for Individualized Infection Medicine (CiiM), Hannover Medical School, Hannover, Germany; 7 Department of General Practice, University Medical Center Göttingen, Göttingen, Germany; 8 Department of Family Medicine, Michigan State University, Grand Rapids, MI USA; 9 Biostatistics Research Group, Helmholtz Centre for Infection Research, Brunswick, Germany

**Keywords:** AGEs, COVID-19, GC–MS, MDA, NO, ROC, Symptoms

## Abstract

In this study, we investigated the status of amino acids, their post-translational modifications (PTM), major nitric oxide (NO) metabolites and of malondialdehyde (MDA) as a biomarker of oxidative stress in serum and urine samples of long COVID (LoCo, *n* = 124) and ex COVID (ExCo, *n* = 24) human subjects collected in 2022. Amino acids and metabolites were measured by gas chromatography–mass spectrometry (GC–MS) methods using stable-isotope labelled analogs as internal standards. There were no differences with respect to circulating and excretory arginine and asymmetric dimethylarginine (ADMA). LoCo participants excreted higher amounts of guanidino acetate than ExCo participants (17.8 ± 10.4 µM/mM vs. 12.6 ± 8.86 µM/mM, *P* = 0.005). By contrast, LoCo participants excreted lower amounts of the advanced glycation end-product (AGE) *N*^G^-carboxyethylarginine (CEA) than ExCo participants did (0.675 ± 0.781 µM/mM vs. 1.16 ± 2.04 µM/mM, *P* = 0.0326). The serum concentrations of MDA did not differ between the groups, indicating no elevated oxidative stress in LoCo or ExCo. The serum concentration of nitrite was lower in LoCo compared to ExCo (1.96 ± 0.92 µM vs. 2.56 ± 1.08 µM; AUC, 0.718), suggesting altered NO synthesis in the endothelium. The serum concentration of nitrite correlated inversely with the symptom anxiety (*r* = − 0.293, *P* = 0.0003). The creatinine-corrected urinary excretion of Lys and its metabolite L-5-hydroxy-Lys correlated positively with COVID toes (*r* = 0.306, *P* = 0.00027) and sore throat (*r* = 0.302, *P* = 0.0003). Our results suggest that amino acid metabolism, PTM and oxidative stress are not severely affected in long COVID. LoCo participants may have a lower circulating NO reservoir than ExCo.

**Supplementary Information:**

The online version contains supplementary material available at 10.1007/s00726-023-03305-1.

## Introduction

The coronavirus disease 2019 (COVID-19) is an infectious disease caused by severe acute respiratory syndrome coronavirus 2 (SARS-CoV-2). The sequelae of COVID-19 can last several weeks or months. This phenomenon is called the post-acute sequelae of COVID-19 (PASC) or long COVID-19 (LoCo). SARS-CoV-2 infection causes direct damage to multiple organs. With varying symptoms, LoCo also influences physical and cognitive function, quality of life, and everyday activity (Yuan et al. 2023, Schröder et al. 2022). The systemic, multi-organ feature of COVID-19 prompted investigations on SARS-CoV-2 infection using metabolomic studies (Bruzzone et al. 2023). Metabolomic and lipidomic studies revealed that various pathways might be differently affected by COVID-19 (Bruzzone et al. 2023). These studies revealed that the concentration of metabolites in plasma, serum and very few in urine correlated with the severity of acute COVID-19 and could, therefore, be used as biomarkers for this disease or to investigate drug effects and recovery (Bruzzone et al. 2023). Metabolites, which were identified to be altered in COVID-19, include the amino acids arginine (Arg), glutamate (Glu) and phenylalanine (Phe).

**Fig. 1:**
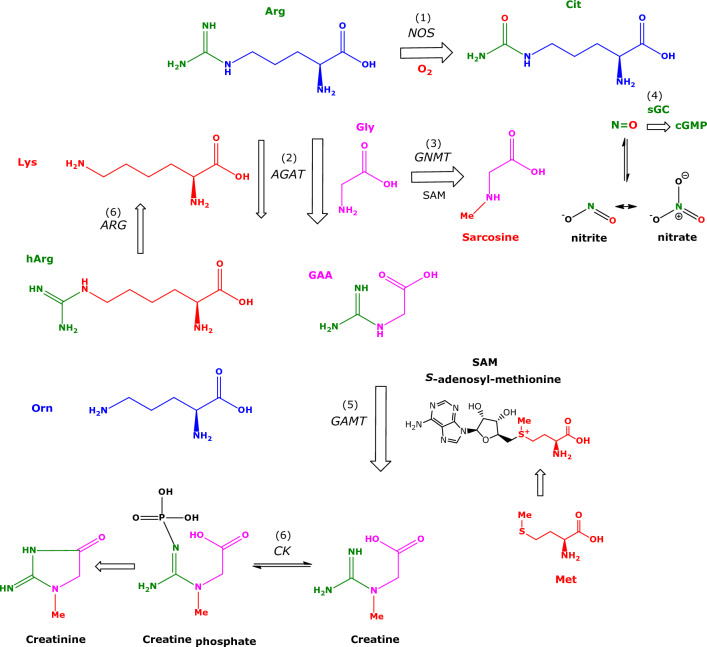
Simplified schematic presentation of two L-arginine-involving pathways and their metabolites. L-Arginine (Arg) is the common substrate for many enzymes as indicated by numbers in parentheses. (1) Nitric oxide synthase (NOS) isoforms convert Arg to L-citrulline (Cit) and nitric oxide (NO). (4) NO activates soluble guanylyl cyclase (sGC) to produce its second messenger cyclic guanylyl monophosphate (cGMP). NO is a potent vasodilatator and inhibitor of platelet aggregation, and a neurotransmitter. NO is oxidized to the interconvertible nitrite and nitrate. Nitrite and nitrate are useful measures of NOS activity and a pool of NO activity in human body. (2) The enzyme arginine:glycine amidinotransferase (AGAT) catalyzes the formation of L-homoarginine (hArg) and guanidinoacetate (GAA), which is the direct precursor of creatine via methylation of GAA by guanidinoacetate methyltransferase (GAMT) (5). (3) Glycine (Gly) is methylated by glycine-*N*-methyltransferase (GNMT) to sarcosine (*N*-methylglycine). (6) Arginase (ARG) hydrolyses Arg to ornithine (Orn), and hArg to L-lysine (Lys). *S*-Adenosylmethionine (SAM) is the common cofactor, the methyl group (Me) donor, of GAMT and GNMT. Arg residues in proteins are methylated and subsequently proteolyzed to form the monomethyl Arg (LMMA) and the asymmetric dimethyl Arg (ADMA). LMMA and ADMA are endogenous inhibitors of NOS activity (not shown). (6) Creatine/creatine phosphate are energy-related molecules. CK, creatine kinase

Amino acids are involved in many pathways and play numerous important roles in humans. L-Arginine (Arg) is one of the most versatile amino acids in the human body (Wu and Morris 1998). Free Arg is a precursor for numerous important biomolecules such as nitric oxide (NO) and guanidino acetate (GAA), the latter being the precursor of the energy-related creatine (Fig. 1). Arg residues in proteins undergo several post-translational modifications (PTM), notably guanidine (*N*^G^) methylation and citrullination (Tsikas 2021). Proteolysis of *N*^G^-methylated proteins releases monomethylarginine (LMMA), asymmetric dimethylarginine (ADMA) and symmetric dimethylarginine (SDMA). These methylated Arg metabolites inhibit the activity of NO synthase (NOS) isoforms which convert Arg to NO and L-citrulline (Cit) (Fig. 1). NO is a gaseous short-lived free radical molecule, a neurotransmitter, and one of the most potent endogenous vasodilators and inhibitors of platelet aggregation (Tsikas 2008). Under physiological conditions, Arg-consuming pathways change dynamically but are in well balanced through tightly cellular interactions. Valid analytical methods are required to determine deviations from “normal” values and intervals of certain metabolites in the blood, which may then be used in diagnosis and therapy. The determination of several biochemical parameters enables a more complex assessment of the imbalance caused by a disease or by other circumstances (Tsikas 2022). In Arg-involving pathways, the Arg bioavailability is of key importance and is dependent on many factors including its cellular transport (Kittel and Maas 2014) and the arginase (ARG) activity (Caldwell et al. 2018). Expression and activity of ARG is generally thought to be of particular importance in NOS-expressing compartments. However, recent studies question a direct competition between ARG and NOS for the common substrate Arg (Becker et al. 2009; Momma et al. 2022; Tsikas and Büttner 2023).

AGEs are glycated amino acids, specifically L-lysine (Lys), Arg, and L-cysteine (Cys). The receptor of AGEs, i.e., RAGE, can interact with AGEs and various other ligands in the lung and in the heart, activating diverse cellular signaling pathways (Twarda-Clapa et al. 2022). RAGE-mediated effects such as oxidative stress, inflammatory responses, proliferation, and apoptosis (Xie et al. 2013) may contribute to extensive tissue damage observed in acute SARS-CoV2 (Roy et al. 2020).

In consideration of the paramount significance of NO and oxidative stress in health and disease (Giustarini et al. 2009; Tsikas 2017), the aim of the present study was to characterize the status of the Arg/NO pathway, of amino acids involved in other pathways, and of oxidative stress in long COVID (LoCo) compared to ex COVID (ExCo) subjects. Participants were recruited through the DEFEAT Corona study, a joint project, which aims at characterizing long COVID (LoCo) in a population-based sample (Mikuteit et al. 2022). Native and modified amino acids produced by catabolism and PTM including methylation and glycation were analyzed in serum and urine samples of LoCo and ExCo humans. We hypothesized that there will be a difference between LoCo and ExCo participants with respect to circulating and urinary concentrations of the Arg/NO pathway, other amino acids-involving pathways, as well to malondialdehyde (MDA) as a biomarker of oxidative stress. We also hypothesized that recovery from the disease would result in associations of measured concentrations with the severity of long COVID symptoms.

## Methods

### Participants

In previous studies, we investigated the long-term consequences of COVID-19 and the pandemic and reported a protocol of the Web-Based, Longitudinal Observational Study (DEFEAT) (Mikuteit et al. 2022). The present work is part of this study. The audiological profile and self-reported tinnitus and vertigo or dizziness of adult long-COVID participants have been recently reported (Degen et al. 2022a, b).

The study is registered in the German clinical trial registry (DRKS00026007) and has been approved by the institutional review board of both Hannover Medical School (9948_BO_K_2021) and University Medical Center Göttingen (29/3/21). Participants were recruited via newspaper announcements, home pages, posters, and flyers in regional, general practices, or long COVID self-support groups, in the outpatient clinics of Hannover Medical School and University Medical Center Göttingen, through the cooperation partners and local public health authorities. Inclusion criteria were people aged 18 years or older, the provision of an informed consent, and the information on whether they had COVID-19 (with or without sequelae). We included participants who answered the baseline questionnaire and whose COVID-19 infection was longer that 4 weeks ago. Exclusion criteria were being younger than 18 years, refusal or inability to provide an informed consent or not residing currently in Germany. Study participation was voluntary, and participants had the right to withdraw consent at any time and without disclosure of reasons for withdrawal. Participants received written information on study procedures and data management, before providing informed consent for each study step. All collected data were pseudonymized and stored safely on servers of the Hannover Medical School or the University of Medical Center Göttingen. Participants considered in the present work did not follow any dietary restrictions prior to the sampling that took place between 14 January 2022 and 6 February 2022 (8 a.m. and 2 p.m.). Metabolites in serum and urine samples of 148 participants were analyzed by gas chromatography–mass spectrometry (GC–MS) in July and August 2022 as described below.

**Fig. 2:**
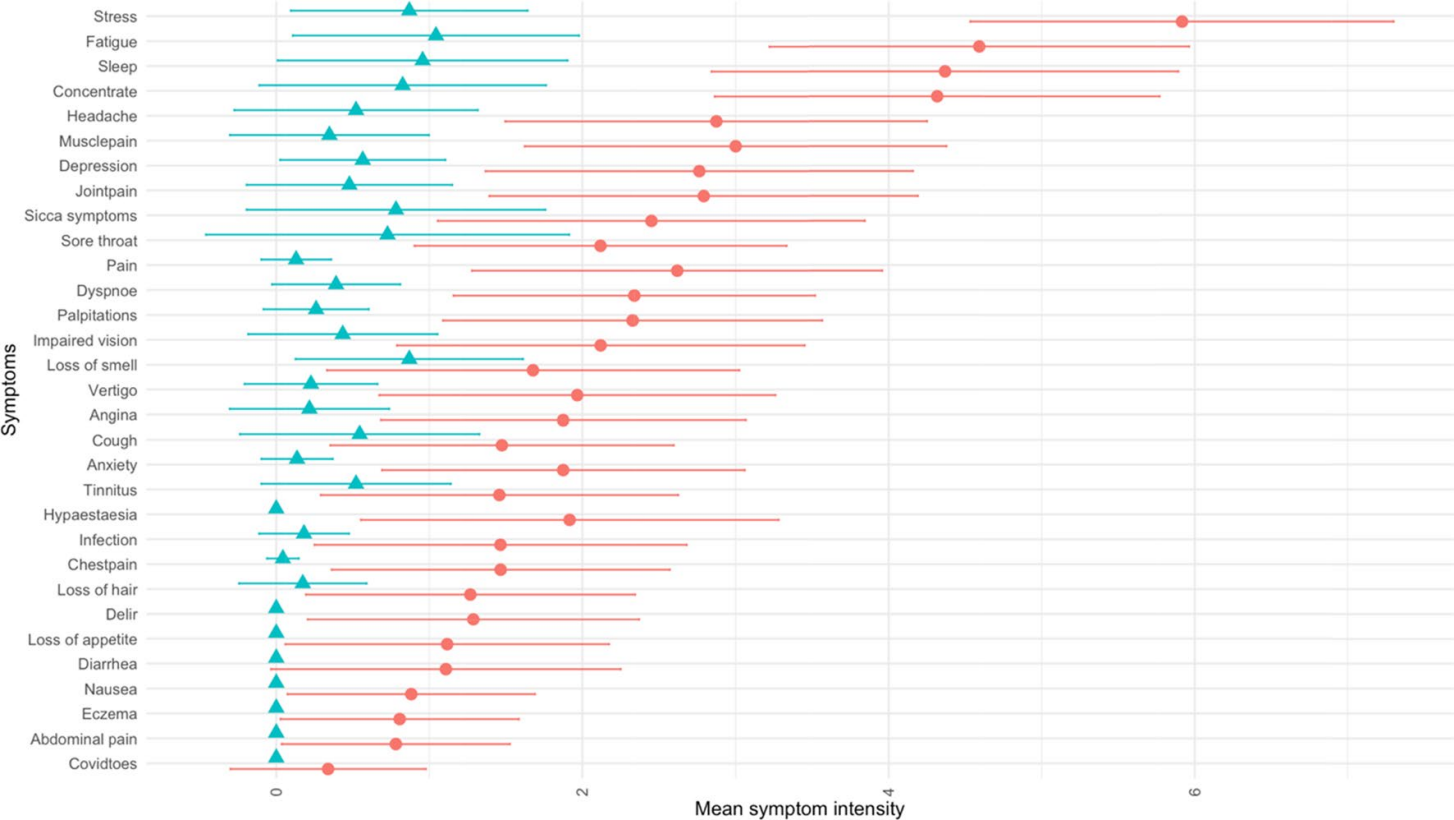
Self-perceived intensity of symptoms (mean with corresponding standard deviation as error bars) in the LoCo (red circles) and ExCo (blue triangles) groups. There were significant differences of the severity of the symptoms between the two groups for the symptoms dyspnea, palpitation, angina pectoris, chest pain, fatigue, pain, lack of concentration, headache, sleep disturbances, paresthesia, vertigo, joint and muscle pain, depression and anxiety (student’s *t*-test, *P*-values corrected with Bonferroni-Holm method)

The comprehensive questionnaire comprised items on sociodemographic, self-perceived health status (EQ-5D-3L; Rabin and Charro 2001), SARS-CoV-2 infection and the progress of the disease including possible late symptoms. The list of symptoms was adapted from the World Health Organization Case Report Form and the UK Long COVID guideline and participants rated severity on a scale from 0 to 10, where 0 indicated no symptom at all and 10 the highest symptom burden thinkable (Shah et al. 2021) (Fig. 2). The participants were divided into two groups, i.e., one group with new or ongoing symptoms after acute COVID-19 for at least 4 weeks (long COVID, LoCo) and one group with a former COVID-19 episode but without long-term symptoms (ex COVID, ExCo) depending on their self-assessment. Participants were invited to an appointment at our studies’ outpatient clinic, where present symptoms were assessed and blood were drawn and urine (spontaneous) samples were collected. The blood was analyzed at the Hannover Medical School for estimated glomerular filtration rate (eGFR) and serum C-reactive protein (sCRP). Heart failure, coronary heart disease, atrial fibrillation, cardiac arrhythmia, and peripheral arterial disease were assigned as cardiovascular diseases. Diabetes mellitus (type 1 and 2), gout, biliary diseases, obesity and thyroid diseases were classified as metabolic diseases. Asthma and chronic obstructive pulmonary diseases were rated as pneumological comorbidities. Hepatitis, HIV, inflammatory bowel disease, psoriasis, rheumatologic diseases, polymyalgia rheumatica, neurodermatitis and other autoimmune diseases were subsumed as inflammatory and autoimmune diseases. Eventually, dementia, depression, schizophrenia, migraine, epilepsy and Parkinson’s disease were rated as neuropsychiatric comorbidities.

### Biochemical analyses

The concentrations of all analytes presented in the study were determined by previously reported validated gas chromatography–mass spectrometry (GC–MS) methods using stable-isotope labelled analogs as internal standards. We measured in serum and urine samples several members of the Arg/NO pathway, including Arg, the substrate of NOS, ADMA, an endogenous inhibitor of NOS, and nitrite and nitrate, major metabolites of NO. We also measured members of other Arg-involving pathways, including GAA and sarcosine (Sarc) which is a metabolite of Gly. Malondialdehyde (MDA) was measured as a biomarker of oxidative stress, notably of lipid peroxidation (Tsikas 2017). With regard to the possible involvement of AGEs and RAGE, the urinary excretion of AGEs was also examined.

Specifically, nitrite, nitrate, creatinine and MDA were measured simultaneously in 100-µL aliquots of serum or urine samples using ^15^N-labeled nitrite and nitrate, and ^2^H-labeled creatinine and MDA for these analytes (Tsikas 2000; Hanff et al. 2017). Amino acids and their metabolites from post-translational modifications (PTM) including methylation and glycation were measured each in 10-µL aliquots of serum or urine using in situ prepared ^2^H-labeled methyl esters of the individual amino acids (Baskal et al. 2021a). The urinary excretion rates of amino acids were corrected for the urinary excretion rate of creatinine, and these results are presented as µM analyte per mM creatinine. The equilibrium constant for the AGAT-catalyzed formation of GAA (i.e., *K*_GAA_) from L-arginine (Arg) and glycine (Gly) was determined as described elsewhere (Tsikas 2022). The equilibrium constant for the AGAT-catalyzed formation of hArg (i.e., *K*_harg_) from Arg and L-lysine (Lys) was determined similarly (Tsikas 2022). Because of co-elution and interconversion (Hanff et al. 2019), the sum concentration for the following amino acids is reported: leucine and isoleucine (Leu + Ile), glutamate and glutamine (Glu + Gln), ornithine and citrulline (Orn + Cit), aspartate and asparagine (Asp + Asn). For 5-hydroxy-lysine (5-OH-Lys), we observed two baseline-separated GC–MS peaks which were assigned to L-5-OH-Lys (L-5OH-Lys) and D-5-OH-Lys (D-5OH-Lys) (Baskal et al. 2021a, b). 4-Hydroxy-proline was observed as a single GC–MS peak and was assigned to *trans*-4-hydroxy-proline (OH-Pro) (Hanff et al. 2019). OH-Pro and 5-OH-Lys are produced by enzymatic hydroxylation of Pro and Lys residues in various proteins including collagen (Salo and Myllyharju 2021). It should be noted that some metabolites were measurable in urine, but not in serum and reversely.

Fractional excretion (FE, %) values were calculated for all analytes by dividing the concentration ratio of creatinine (Crea) in serum (S) and urine (U), i.e., [Crea]_S_/[Crea]_U_, by the concentration ratio of an amino acid (AA) in serum and urine, i.e., [AA]_S_/[AA]_U_, measured at a certain time point, and by multiplying the result by 100 (see Formula F1).

F1 $${\text{FE }}\left( \% \right) \, = \, \left( {\left[ {{\text{Crea}}} \right]_{{\text{P}}} /\left[ {{\text{Crea}}} \right]_{{\text{U}}} } \right) \, / \, \left[ {{\text{AA}}} \right]_{{\text{P}}} /\left[ {{\text{AA}}} \right]_{{\text{U}}} ) \times {1}00\,\,\%$$

Study samples were analyzed alongside quality control (QC) samples to determine the precision and the accuracy of the GC–MS methods for the analytes in the serum and urine study samples. For the simultaneous analysis of the amino acids (Baskal et al. 2021a, b), the urine sample #2 and the serum sample #140 were randomly assigned as QC samples and were analyzed in quintuplicate. For the simultaneous analysis of creatinine, nitrate, nitrite and MDA (Hanff et al. 2017), the above-mentioned QC samples were analyzed in duplicate, in unspiked (QC1) and in spiked samples (QC2, QC3) with the spiked analytes being in relevant concentration ranges (Tsikas 2009). The precision of the GC–MS method was calculated as the relative standard deviation (RSD, %). The accuracy of the GC–MS method for creatinine, nitrate, nitrite and MDA was determined as recovery (%) for added concentrations after subtraction of the baseline concentrations and multiplying the outcome by 100. The results of the QC samples are reported in the supplementary Table S1. All precision and recovery values were within acceptable ranges (Tsikas 2009).

### Statistical analyses

Statistical analyses were performed on the full data set, which consisted of 33 targeted metabolites measured in 296 samples.

Data were processed using SAS^®^ Studio (SAS Institute Inc, Cary, NC), STATA 14 (StataCorp, College Station, TX, USA) and RStudio (version 4.1.2, with packages tidyverse (version 1.3.2), ggpubr (version 0.6.0), emmeans (version 1.8.2) and Hmisc (version 5.0–1)). Graphs were plotted using GraphPad Prism 7 (Graph Pad Software, San Diego, CA) and RStudio. Data normality was tested by the D’Agostino and Pearson normality test. Data are reported as means ± standard deviation (SD) or median with interquartile range (IQR). Comparison of groups was conducted using Mann–Whitney U test, Kruskal Wallis test, student’s t test or one-way ANOVA (estimated marginal means as post-hoc tests), as appropriate. Area under the curve (AUC) values were determined by receiver operating characteristic (ROC). Pearson’s and Spearman’s rho was used to analyze correlations between metabolites and symptom strength, where appropriate. Statistical significance was determined as *P* < 0.05, if not otherwise stated. For the comparison and correlation analyses, *P* values were adjusted using Bonferroni-Holm correction, and Tukey correction for post-hoc testing after ANOVA.

## Results

### Study sample

The characteristics of the investigated participants are summarized in Table 1 and in the Supplement to this work. Figure 2 shows the symptoms in the LoCo and ExCo. The groups differed with respect to gender, the intensity of the symptoms, self-perceived health status, time since infection and for some comorbidities. The gravest symptoms were stress, fatigue, lack of concentration and sleep disturbances in the LoCo subjects.

**Table 1:** Demographic and clinical characteristics of the long COVID (LoCo) and ex COVID (ExCo) participants of the study. Data are shown as mean with standard deviation (SD)

Parameter	All	LoCo	ExCo	*P* value
Number of subjects	148	124	24	
Age (*y*)	43.0 (12.0)	42.7 (11.8)	44.8 (12.8)	0.432^b^
*Gender* (*n*, %)				0.015^c^
Females	112 (75.7)	99 (79.8)	13 (54.2)	
Males	36 (24.3)	25 (20.2)	11 (45.8)	
Time since acute infection (d)	362 (166)	391 (143)	126 (147)	< 0.0001^b^
Hospitalization (*n*, %; *n* = 131)	11 (8.4)	11 (9.4)	0 (0.0)	0.6064^d^
eGFR (mL/h)	n.m	93.5 (16.6)	n.m	n.a
sCRP (mg/L)	n.m	3.31 (5.53)	n.m	n.a
Heart rate (bpm;* n* = 120)		81 (58)		
EQ-5D Visual analogue scale ^a^ (points)	60.4 (23.7)	56.1 (21.9)	81.8 (20.9)	< 0.001^b^
*Comorbidities* (*n*, %)				
Cardiovascular	25 (18.5)	23 (19.2)	2 (15.4)	1.000^c^
Metabolic	40 (29.6)	38 (31.7)	2 (15.4)	0.369^c^
Lung	18 (13.3)	16 (13.3)	2 (15.4)	1.000^c^
Chr. inflam. and autoimmune	13 (9.6)	11 (9.2)	2 (15.4)	0.822^c^
Neuropsychiatric	36 (26.7)	36 (30.0)	0 (0.0)	0.030^c^
Other	37 (27.4)	37 (30.8)	0 (0.0)	0.027^c^

*eGFR* sCRP and heart rate were not available for the ExCo group, *eGFR* estimated glomerular filtration rate, *sCRP* serum C-reactive protein

^a^Self-perceived health status measured by the visual analogue scale of EQ-5D scale from 0 to 100, with 100 being the best status

^b^Students t-test

^c^Chi square test

^d^Fisher exact test

The results of the study are summarized and illustrated in the following Tables and Figures. The concentrations of the measured analytes in the serum samples of the LoCo and ExCo participants of the study are summarized in Table 2. This Table also lists the results of the statistical analyses between the two groups. Statistically significant differences were found between LoCo and ExCo for the serum concentrations of the Gly metabolite sarcosine, i.e., Sarc, (0.877 ± 0.313 µM vs. 1.22 ± 0.532 µM, respectively, *P* = 0.0002) and of the Arg-derived NO metabolite nitrite (1.96 ± 0.919 µM vs. 2.56 ± 1.08 µM, respectively, *P* = 0.0006). ExCo participants had higher mean serum concentrations of Sarc (by 28%) and nitrite (by 23%). These findings remained significant after correction (adjustment) of the *P*-value. Borderline significances (*P* < 0.1) between LoCo and ExCo were observed for the serum concentrations of the branched-chain amino acids Leu + Ile (LoCo < ExCo), for the Arg metabolites Orn + Cit (LoCo < ExCo), and for nitrate (LoCo > ExCo), the major NO metabolite (Table 2).

**Table 2:** Serum concentrations (in µM) of the measured analytes in the long COVID (LoCo, *n* = 124) and ex COVID (ExCo, *n* = 24) participants of the study and their statistical analysis

Analyte	LoCo Mean (SD)	ExCo Mean (SD)	P (M-W)	*P* adj	AUC (SE) LoCo vs ExCo	*P* (ROC)	*P adj. (ROC)*
Amino acids and PTMs							
Ala	344 (93.3)	345 (82.7)	0.7049	1.0	0.525 (0.061)	0.7022	1.0
Asp + Asn	83.4 (18.3)	87.4 (28.4)	0.3826	1.0	0.529 (0.070)	0.6509	1. 0
Glu + Gln	727 (153.6)	767 (188.1)	0.2673*	1.0	0.553 (0.069)	0.4141	1.0
Phe	68.3 (15.5)	72.6 (15.4)	0.2155*	1.0	0.567 (0.063)	0.2982	1.0
Gly	224 (60.6)	239 (72.5)	0.4010	1.0	0.555 (0.068)	0.3980	1.0
Leu + Ile	193.3 (52.5)	221 (71.9)	0.0943	1.0	0.608 (0.066)	0.09398	1.0
Lys	162.5 (46.5)	169.7 (52.5)	0.5024*	1.0	0.556 (0.069)	0.3850	1.0
Met	63.4 (9.34)	66.2 (10.9)	0.2069*	1.0	0.575 (0.067)	0.2440	1.0
Pro	157.4 (57.9)	177.5 (69.9)	0.1837	1.0	0.586 (0.065)	0.1822	1.0
Arg	90.9 (23.1)	88.2 (37.9)	0.2109	1.0	0.581 (0.064)	0.2091	1.0
Ser	138.2 (28.7)	139.5 (32.3)	0.8359*	1.0	0.501 (0.070)	0.9938	1.0
Thr	137.2 (38.7)	142.1 (36.7)	0.5753*	1.0	0.570 (0.062)	0.2804	1.0
Val	272 (76.3)	294 (79.0)	0.1515	1.0	0.593 (0.065)	0.1504	1.0
Tyr	47.3 (14.8)	51.7 (15.8)	0.2090	1.0	0.582 (0.062)	0.2072	1.0
Sarc	0.877 (0.313)	1.22 (0.532)	0.0002	0.0052	0.732 (0.063)	0.0003	0.0078
Orn + Cit	91.0 (29.1)	105.8 (39.9)	0.0948	1.0	0.608 (0.066)	0.09449	1.0
GAA	1.99 (0.424)	1.90 (0.590)	0.2006	1.0	0.583 (0.070)	0.1989	1.0
hArg	1.43 (0.570)	1.37 (0.670)	0.5493	1.0	0.539 (0.072)	0.5462	1.0
OH-Pro	6.62 (3.21)	6.94 (4.15)	0.9804	1.0	0.502 (0.068)	0.9792	1.0
D-5OH-Lys	0.323 (0.054)	0.317 (0.051)	0.5742*	1.0	0.526 (0.063)	0.6868	1.0
L-5OH-Lys	0.859 (0.163)	0.825 (0.197)	0.1295	1.0	0.598 (0.064)	0.1288	1.0
MML	4.90 (3.98)	4.71 (3.90)	0.8880	1.0	0.509 (0.059)	0.8862	1.0
Creatinine–Nitrate–Nitrite–MDA							
Creatinine	104.3 (16.8)	105.9 (17.4)	0.6825*	1.0	0.547 (0.062)	0.4633	1.0
Nitrate	67.2 (18.1)	64.2 (31.0)	0.0678	1.0	0.618 (0.064)	0.06792	1.0
Nitrite	1.96 (0.919)	2.56 (1.08)	0.0006	0.0150	0.718 (0.054)	0.0007	0.0175
MDA	1.10 (0.377)	1.11 (0.215)	0.2175	1.0	0.580 (0.058)	0.2157	1.0000

*SD* standard deviation, *SE* standard error, *AUC* area under the curve, *ROC* receiver operating characteristic, *M-W* Mann–Whitney U test, *unpaired* t* test

The concentration of urinary creatinine and the creatinine-corrected excretion rates of the analytes measured in the urine samples of the LoCo and ExCo participants of the study are summarized in Table 3. This Table also lists the results of the statistical analyses between the two groups. Statistically significant differences were found between LoCo and ExCo for the creatinine-corrected excretion rates of serum concentrations of the Gly metabolites sarcosine, i.e., Sarc (0.219 ± 0.103 µM/mM vs. 0.299 ± 0.202 µM/mM, respectively, *P* = 0.0221) and guanidinoacetate, i.e., GAA (17.8 ± 10.4 µM/mM vs. 12.6 ± 8.86 µM/mM, respectively, *P* = 0.005). Statistically significant differences were also found for the Lys metabolite D-5OH-Lys (0.0835 ± 0.0492 µM/mM vs. 0.100 ± 0.0493 µM/mM, respectively, *P* = 0.0333), and the advanced glycation end-product (AGE) of *N*^G^-carboxyethyl-L-arginine, i.e., CEA (0.675 ± 0.781 µM/mM vs. 1.16 ± 2.04 µM/mM, respectively, *P* = 0.0326) (Table 3). ExCo participants had higher mean excretion rates of Sarc (by 27%), D-5OH-Lys (by 17%) and CEA (by 14%). In contrast, LoCo participants had higher mean excretion rates of GAA (by 42%) compared to ExCo participants. Borderline significances (*P* < 0.1) between LoCo and ExCo were observed for the mean excretion rates of Gly and hArg. After *P*-value correction, these differences were not significant.

**Table 3:** Urinary creatinine concentration (in mM) and creatinine-corrected excretion rates (in µM/mM) of the measured analytes in the long COVID (LoCo, *n* = 124) and ex COVID (ExCo, *n* = 24) participants of the study and their statistical analysis

Analyte	LoCo Mean (SD)	ExCo Mean (SD)	*P* (M-W)	*P* adj	AUC (SE) LoCo vs ExCo	*P* (ROC)	*P adj. (ROC)*
Amino acids and PTMs							
Ala	14.8 (9.1)	15.7 (6.2)	0.1257	1.0	0.599 (0.060)	0.1249	1.0
Asp + Asn	11.9 (5.1)	12.4 (4.8)	0.6991	1.0	0.525 (0.062)	0.6964	1.0
Glu + Gln	65.4 (25.1)	70.4 (20.9)	0.2099	1.0	0.581 (0.060)	0.2081	1.0
Phe	3.39 (1.3)	3.59 (1.1)	0.3753	1.0	0.558 (0.062)	0.3723	1.0
Gly	88.7 (72.6)	106.3 (78.2)	0.0789	1.0	0.614 (0.057)	0.0788	1.0
Leu + Ile	9.71 (9.09)	13.6 (17.4)	0.1889	1.0	0.585 (0.068)	0.1873	1.0
Lys	8.69 (9.92)	6.43 (4.46)	0.5898	1.0	0.535 (0.055)	0.5867	1.0
Met	5.67 (1.90)	5.79 (1.77)	0.7951	1.0	0.517 (0.058)	0.7928	1.0
Pro	1.16 (0.42)	1.89 (3.28)	0.2816	1.0	0.570 (0.066)	0.2792	1.0
Arg	1.52 (0.60)	1.34 (0.46)	0.1132	1.0	0.603 (0.062)	0.1127	1.0
Ser	23.0 (10.3)	25.4 (8.8)	0.1034	1.0	0.605 (0.058)	0.1030	1.0
Thr	14.4 (8.2)	14.2 (4.7)	0.4068	1.0	0.554 (0.058)	0.4038	1.0
Val	3.68 (1.31)	3.47 (0.91)	0.4663	1.0	0.547 (0.058)	0.4632	1.0
Tyr	9.03 (3.29)	8.90 (2.24)	0.9105	1.0	0.507 (0.060)	0.9089	1.0
Sarc	0.219 (0.103)	0.299 (0.202)	0.0221	0.685	0.647 (0.056)	0.0229	0.710
Orn + Cit	2.57 (0.23)	2.82 (0.96)	0.2504	1.0	0.575 (0.062)	0.2482	1.0
GAA	17.8 (10.4)	12.6 (8.9)	0.0050	0.160	0.680 (0.060)	0.0054	0.174
hArg	0.165 (0.253)	0.0842 (0.086)	0.0766	1.0	0.614 (0.055)	0.07657	1.0
OH-Pro	0.280 (0.280)	0.327 (0.292)	0.8371	1.0	0.513 (0.068)	0.8352	1.0
D-5OH-Lys	0.084 (0.050)	0.100 (0.049)	0.0333	0.978	0.637 (0.063)	0.0339	0.996
L-5OH-Lys	0.377 (0.165)	0.374 (0.222)	0.3153	1.0	0.565 (0.060)	0.3129	1.0
MML	0.707 (0.812)	0.607 (0.605)	0.8050	1.0	0.516 (0.063)	0.8028	1.0
ADMA	2.20 (0.67)	2.28 (0.65)	0.6315	1.0	0.531 (0.070)	0.6286	1.0
CML	0.577 (0.304)	0.572 (0.249)	0.7010	1.0	0.525 (0.064)	0.6984	1.0
CEL	0.463 (0.212)	0.468 (0.222)	0.9577	1.0	0.504 (0.064)	0.9564	1.0
CEA	0.675 (0.781)	1.16 (2.04)	0.0326	0.978	0.638 (0.064)	0.0332	0.996
CEC	0.568 (0.490)	0.610 (0.419)	0.3965	1.0	0.555 (0.063)	0.3936	1.0
Furosine	0.071 (0.051)	0.065 (0.041)	0.7811	1.0	0.518 (0.062)	0.7788	1.0
Hypusine	0.181 (0.052)	0.174 (0.045)	0.7372	1.0	0.522 (0.060)	0.7353	1.0
Creatinine–Nitrate–Nitrite							
Creatinine	10.54 (9.51)	11.3^#^ (9.2)	0.7068	1.0	0.525 (0.067)	0.7041	1.0
Nitrate	78.60 (50.0)	79.1 (86.2)	0.2255	1.0	0.579 (0.064)	0.2235	1.0
Nitrite	0.362 (0.559)	0.287 (0.222)	0.4067	1.0	0.554 (0.068)	0.4038	1.0

*SD* standard deviation, *SE* standard error, *AUC* area under the curve, *ROC* receiver operating characteristic, *M-W* mann–whitney U test, *P* values adjusted with Bonferroni-Holm method

As our cohort consisted of female and male participants, we examined potential effects of the gender on the serum concentrations (Table S2) and the urinary excretion rates (Table S3) of the measured analytes. In fact, gender-related statistical differences were for analytes in serum and in urine. The highest difference was observed for serum creatinine (99.9 ± 15.2 µM in females vs. 119.3 ± 12.8 µM in males, *P* < 0.0001) (Table S2). A high difference was also observed for urinary creatinine (9.92 ± 9.04 mM in females vs. 13.0 ± 10.4 mM in males, *P* = 0.0453) (Table S3), but it should be noted that urine was collected by spontaneous micturition. In serum, gender-related differences were found for Leu + Ile, Lys, Pro, Val, Orn + Cit, hArg, OH-Pro, and MDA. The serum concentrations of these analytes were higher in the males (Table S2). Statistically significant gender-related differences were observed for the creatinine-corrected urinary excretion rates of many analytes, which were higher in the females (Asp + Asn, Gly, Met, GAA, L-5OH-Lys, ADMA, furosine, nitrate and nitrite). Only the creatinine-corrected urinary excretion rate of Pro was higher in the males (Table S3). The fractional excretion rates of the analytes were in normal ranges (Table S4) and did not differ between the LoCo and ExCo groups. Gender-related differences were found for GAA, i.e., the creatine precursor, but not for hArg (Fig. S1). Not all observed differences remained statistically significant after *P*-value correction (Tables S1, S2).

Table 4 summarizes the serum concentrations in LoCo females and LoCo males, as well as in ExCo females and ExCo males. Statistically significant differences (after *P*-value correction) were observed for creatinine (LoCo females vs. LoCo males, *P* < 0.0001; LoCo females vs. ExCo males, *P* = 0.0313; LoCo males vs. ExCo females, *P* = 0.0001); Leu + Ile (LoCo females vs. ExCo males, *P* = 0.0002; ExCo females vs. ExCo males, *P* = 0.0064) and Sarc (LoCo females vs. ExCo females, *P* = 0.0037; LoCo females vs. ExCo males, *P* = 0.0272; LoCo males vs. LoCo females, *P* = 0.0272). For Pro, Orn + Cit, hArg and nitrite, we found differences that were not significant after *P*-value correction.

**Table 4:** Serum concentrations (in µM) in LoCo females (*n* = 99) and LoCo males (*n* = 25), and in ExCo females (*n* = 13) and ExCo males (*n* = 11) of the study

Analyte	LoCo female		LoCo male		ExCo female		ExCo male	
	*n* = 99		*n* = 25		*n* = 13		*n* = 11	
	mean	SD	mean	SD	mean	SD	mean	SD
*Amino acids and PTMs*								
Ala	344	95.8	343	84.7	317	72.0	378	85.3
Asp + Asn	83.4	19.0	83.5	15.6	84.2	30.0	91.1	27.3
Glu + Gln	719	154	760	152.0	729	207	810	161.2
Phe	68.3	16.2	68.5	12.6	70.4	17.1	75.2	13.4
Gly	227	60.8	214	59.7	252	87.5	223	48.8
Leu + Ile^a^	188.2	53.8	213	42.2	188.1	58.8	261	67.7
Lys	159.1	47.5	176.0	40.7	152.4	55.2	190.1	42.9
Met	63.0	9.46	65.2	8.81	63.7	12.3	69.1	8.69
Pro^b^	156.3	60.9	161.9	44.8	150.8	55.7	209	74.2
Arg	92.8	23.8	83.5	19.1	82.9	21.2	94.5	51.8
Ser	139.2	29.4	134.0	25.9	137.0	37.1	142.6	27.1
Thr	138.5	40.1	132.2	32.6	131.9	38.8	154.1	31.5
Val	267	79.6	292	58.8	261	78.0	333	62.9
Tyr	47.8	15.6	45.3	11.6	49.1	19.6	54.7	9.85
Sarc ^c^	0.872	0.322	0.893	0.282	1.24	0.686	1.20	0.292
Orn + Cit^d^	87.9	28.6	103.3	28.3	95.2	38.0	118.5	40.0
GAA	1.96	0.409	2.14	0.460	1.75	0.442	2.06	0.713
hArg^e^	1.39	0.548	1.59	0.632	1.03	0.508	1.78	0.628
OH-Pro	6.25	2.86	8.12	4.07	7.07	5.09	6.78	2.90
D-5OH-Lys	0.319	0.054	0.340	0.052	0.332	0.0510	0.299	0.0459
L-5OH-Lys	0.852	0.162	0.886	0.165	0.898	0.233	0.738	0.0959
MML	5.15	4.10	3.92	3.35	5.34	4.79	3.97	2.52
*Creatinine, Nitrate, Nitrite, MDA*								
Creatinine^f^	99.8	15.0	122.2	10.8	100.1	17.7	112.7	14.9
Nitrate	67.3	17.7	66.7	20.2	70.8	40.8	56.4	9.85
Nitrite^g^	1.95	0.947	1.98	0.814	2.78	1.29	2.31	0.746
MDA	1.08	0.401	1.17	0.254	1.12	0.219	1.09	0.219

ANOVA was performed with Post hoc tests with Tukey correction for *P*-values. Overall *P*-values were corrected with Bonferroni-Holm method

^a^Leu + Ile: LoCo females vs. ExCo males, *P* = 0.0002; ExCo females vs. ExCo males, *P* = 0.0064

^b^Pro: LoCo females vs. ExCo males, *P* = 0.0286

^c^Sarc: LoCo females vs. ExCo females, *P* = 0.0037; LoCo females vs. ExCo males, *P* = 0.0272; LoCo males vs. LoCo females, *P* = 0.0272

^d^Orn + Cit: LoCo females vs. ExCo males, *P* = 0.010

^e^hArg: LoCo males vs. ExCo females, *P* = 0.0257; ExCo females vs. ExCo males, *P* = 0.0092

^f^Creatinine: LoCo females vs. LoCo males, *P* < 0.0001; LoCo females vs. ExCo males, *P* = 0.0313; LoCo males vs. ExCo females, *P* = 0.0001

^g^Nitrite: LoCo females vs. ExCo females, *P* = 0.0184

Table 5 summarizes the urinary excretion rates of the analytes in LoCo females and LoCo males. There were no statistically significant differences after *P*-value correction. Before correction, we found differences for Gly, Pro, Sarc, GAA, ADMA and CEA.

**Table 5:** Urinary creatinine concentration (in mM) and creatinine-corrected excretion rates (in µM/mM) of the measured analytes in the females and males of the LoCo and ExCo groups of the study

Analyte	LoCo female		LoCo male		ExCo female		ExCo male	
	*n* = 99		*n* = 25		*n* = 13		*n* = 11	
	Mean	SD	Mean	SD	Mean	SD	Mean	SD
Amino acids and PTMs								
Ala	15.5	9.54	12.0	6.20	15.6	7.60	15.7	4.38
Asp + Asn	12.4	5.21	10.1	4.03	14.1	5.76	10.4	2.47
Glu + Gln	66.9	25.2	59.2	24.3	76.2	24.4	63.5	13.9
Phe	3.49	1.32	2.99	1.29	3.96	1.24	3.17	0.812
Gly	96.4	77.8	58.1	32.6	128.9	97.1	79.6	36.4
Leu + Ile	10.1	9.54	8.25	7.02	16.6	23.1	10.1	5.35
Lys	8.62	9.93	8.96	10.1	6.77	5.81	6.04	2.23
Met	5.87	1.84	4.86	1.92	6.33	2.16	5.14	0.860
Prol	1.20	0.422	0.970	0.378	1.35	0.427	2.52	4.87
Arg	1.55	0.617	1.37	0.508	1.28	0.370	1.40	0.558
Ser	24.0	10.7	19.2	7.75	28.0	11.0	22.2	3.83
Thr	15.2	8.64	11.4	5.46	14.7	5.54	13.5	3.45
Val	3.79	1.31	3.25	1.25	3.57	1.13	3.36	0.594
Tyr	9.22	3.22	8.32	3.50	9.36	2.40	8.34	2.01
Sarc	0.226	0.108	0.190	0.0778	0.317	0.172	0.277	0.239
Orn + Cit	2.61	0.797	2.41	0.938	3.03	1.21	2.58	0.481
GAA	19.4	11.0	11.7	5.02	14.1	10.5	10.8	6.54
hArg	0.165	0.248	0.164	0.278	0.0988	0.113	0.0669	0.0289
OH-Pro	0.291	0.306	0.236	0.125	0.359	0.281	0.289	0.315
D-5OH-Lys	0.0861	0.0522	0.073	0.0337	0.109	0.057	0.0894	0.0389
L-5OH-Lys	0.388	0.173	0.334	0.117	0.445	0.283	0.289	0.0539
MML	0.706	0.781	0.709	0.943	0.594	0.507	0.622	0.730
ADMA	2.28	0.668	1.90	0.575	2.53	0.696	1.98	0.442
CML	0.589	0.291	0.528	0.353	0.589	0.244	0.552	0.266
CEL	0.475	0.219	0.416	0.178	0.473	0.231	0.461	0.221
CEA	0.614	0.336	0.918	1.61	1.52	2.75	0.717	0.260
CEC	0.563	0.440	0.586	0.660	0.745	0.516	0.450	0.180
Furosine	0.0761	0.0511	0.052	0.0485	0.0669	0.044	0.0626	0.0377
Hypusine	0.183	0.0482	0.174	0.0667	0.168	0.052	0.181	0.0356
Creatinine–Nitrate–Nitrite								
Creatinine	9.81	8.99	13.4	11.1	10.7	9.75	12.1	9.02
Nitrate	83.2	52.9	60.3	30.5	99.0	113.7	55.6	21.4
Nitrite	0.398	0.617	0.220	0.146	0.345	0.272	0.218	0.122

**Fig. 3:**
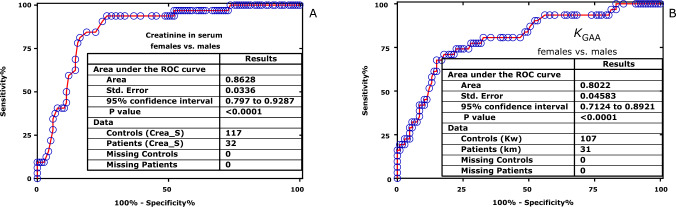
Area under the ROC curves for creatinine (**A**) and for *K*_GAA_ (**B**) in the serum samples of the females and males of the study

There were no differences with respect to the equilibrium constant *K*_GAA_ of the AGAT-catalyzed formation of GAA from Arg and Gly in the LoCo and ExCo groups: *K*_GAA_ = 0.0085 [0.007–0.012] vs. *K*_GAA (ex)_ = 0.0090 [0.00695–0.01205] (Mann–Whitney *U* test, *P* = 0.7301) or the AUC values (0.5233 ± 0.0693, *P* = 0.7283). However, there were differences between females (f) and males (m): *K*_GAA(f)_ = 0.0079 [0.0065–0.0098] vs. *K*_GAA (m)_ = 0.0120 [0.0096–0.0159] (Mann–Whitney *U* test, *P* < 0.0001) and the AUC values (0.8022 ± 0.04583, *P* < 0.0001) (Fig. 3). There were no differences with respect to the equilibrium constant *K*_harg_ of the AGAT-catalyzed formation of hArg from Arg and Lys in the LoCo and ExCo groups: *K*_harg_ = 0.0084 [0.006–0.011] vs. *K*_harg (ex)_ = 0.0073 [0.0055–0.01276] (Mann–Whitney *U* test, *P* = 0.7744) or the AUC values (0.5194 ± 0.0735, *P* = 0.7719). There were differences between females (f) and males (m): *K*_harg(f)_ = 0.00768 [0.00577–0.01031] vs. *K*_harg (m)_ = 0.0120 [0.0096–0.0159] (Mann–Whitney *U* test, *P* < 0.0001) and the AUC values (0.722 ± 0.05446, *P* = 0.0002). Yet, the *K*_GAA(f)_/*K*_harg(f)_ and *K*_GAA(m)_/*K*_harg(m)_ ratios did not differ from each other (1.033 [0.83–1.31] vs 1.05 [0.91–1.51], *P* = 0.4258). These results collaborate with the higher serum creatinine concentrations in the males of our study (Fig. 3).

**Fig. 4:**
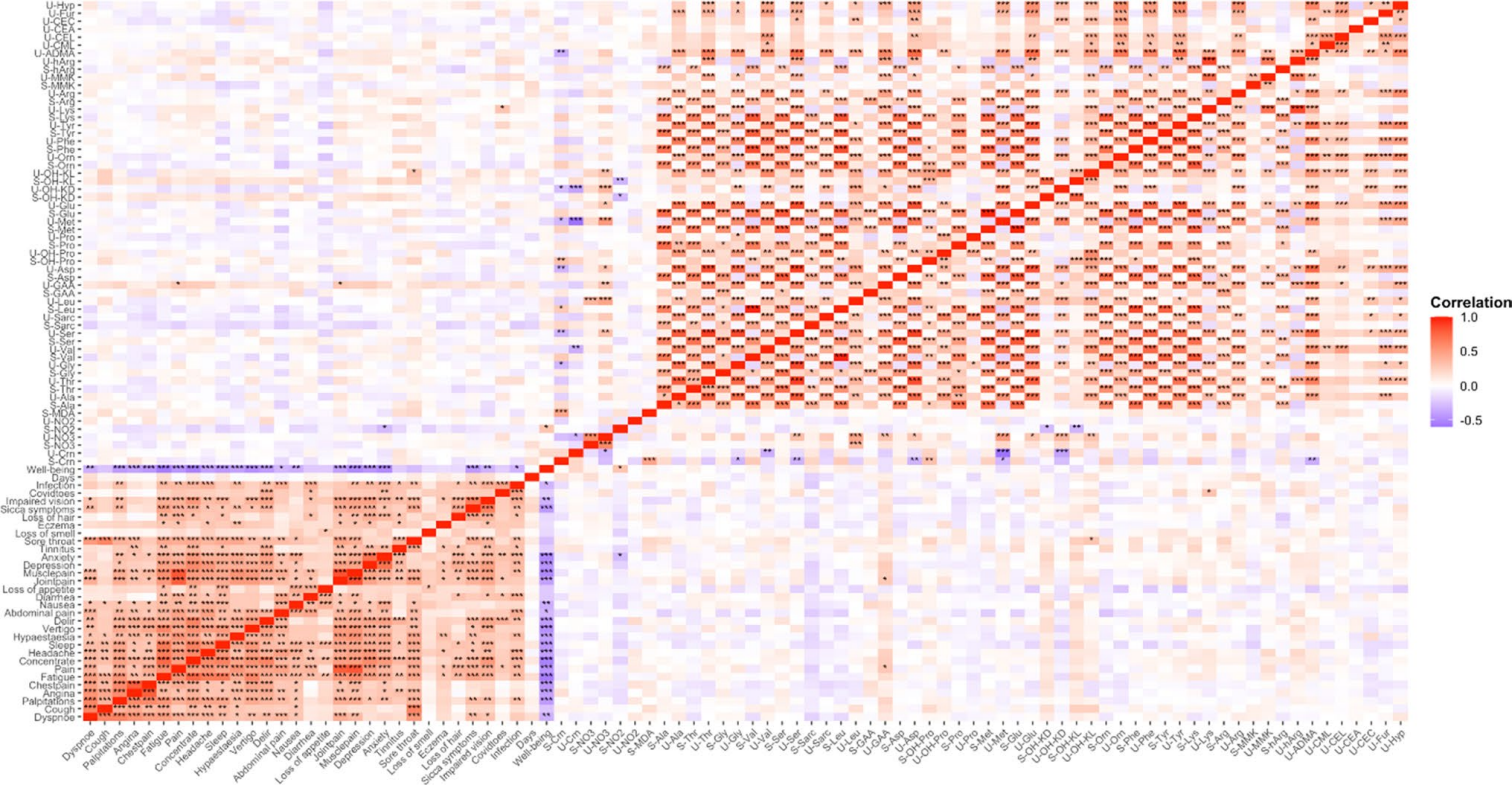
Correlation heatmap between symptoms and concentration of the analytes in the serum (S) and urine (U) samples. The Spearman correlation coefficient *rho* is indicated by color. Significant correlations are indicated as followed: ***, *P* < 0.001; **, *P* < 0.01 and < 0.001; *, *P* < 0.05 and < 0.01. *P*-values are corrected by the Bonferroni-Holm method

We found only few correlations between measured analytes and reported symptoms (Fig. 4). All symptoms noted were not highly pronounced among the participants; for anxiety, mean strength was 1.61 ± 2.27), for sore throat 1.90 ± 2.47 and for COVID toes 0.28 ± 1.18. Thus, serum nitrite concentration correlated negatively with anxiety (*r* = − 0.293, *P* = 0.0003). Creatinine-corrected urinary L-5OH-Lys concentration correlated positively with sore throat (*r* = 0.302, *P* = 0.0003). Creatinine-corrected urinary Lys concentration correlated positively with COVID toes (*r* = 0.306, *P* = 0.00027). The general wellbeing correlated positively with serum nitrite (*r* = 0.303, *P* = 0.025) and negatively with the severity of most symptoms. There were numerous correlations between the analytes in the serum and urine samples and among the symptoms (data not shown).

## Discussion

In the present study, we used validated previously reported GC–MS methods for the quantitative determination of amino acids and their metabolites (Hanff et al. 2017, 2019; Baskal et al. 2021a) in serum and urine samples of human subjects with long COVID (LoCo) and ex COVID (ExCo). The co-processed QC samples indicate that all analytes were measured in the study serum and urine samples with analytically satisfactory precision and accuracy (Table S1). The serum and urinary concentrations of all analytes measured in the study are within ranges reported by our group in previous clinical studies in health and disease using GC–MS (Hanff et al. 2019; Baskal et al. 2021b). They are also in line with concentrations reported by other groups using other analytical methods, including LC–MS (Duranton et al. 2014; Thornalley et al. 2003; Agalou et al. 2005; Thornalley and Rabbani 2014) and other methods including HPLC (Horowitz and Heresztyn 2007; Martens-Lobenhoffer and Bode-Börger 2014; Tsikas 2008; Wang et al. 2021; Kaspar et al. 2008; Waterval et al. 2009; Dai et al. 2014). The serum nitrate and nitrite concentrations measured in post COVID subjects (Wang et al. 2021) are very close to those we measured in the LoCo and ExCo subjects of the present study.

In serum, we found statistical differences for Sarc and nitrite between LoCo and ExCo. Sarc (1.4-fold) and nitrite (1.3-fold) concentrations were higher in ExCo compared to LoCo, and the AUC values of 0.7 were relatively small. In urine, we found statistical differences for Sarc (1.4-fold), GAA (0.7-fold), CEA (1.7-fold) and D-5OH-Lys (1.2-fold) between LoCo and ExCo. The AUC values of 0.6 were even smaller than in serum.

As the gender may influence the homeostasis of amino acids and their metabolites, we conducted sensitivity analyses stratified for male and female gender in the LoCo and ExCo groups accordingly. Expectedly, the highest gender-related difference was observed for creatinine in serum (AUC, 0.863). Differences in serum concentrations of Sarc and nitrite between LoCo and ExCo participants were also present in the female subgroup. This hints to an effect related to the COVID status, and not to gender. The correlation between serum nitrite concentration and general well-being supports possible effects of NO.

With regard to symptoms, serum nitrite concentration was found to correlate indirectly with anxiety symptoms. On the other hand, creatinine-corrected urinary Lys and L-OH-Lys concentrations correlated positively with COVID toes and sore throat, respectively. As the strength of the described symptoms and their correlations were rather weak, their clinical relevance is considered limited.

During our study, a few papers have been reported in the context of COVID-19. In serum analyzed substances included amino acids (Philips and Khan 2021; Páez-Franco et al. 2021; Atila et al. 2021; Fanelli et al. 2022; Ozturk et al. 2022), the NO metabolites nitrite and nitrate (Wang et al. 2021), and the thiobarbituric acid reactive substances (TBARS) as biomarkers of oxidative stress (Vazquez-Agra et al. 2022). In our study, we did not find elevated oxidative stress in the LoCo participants compared to the ExCo subjects, which was measured as MDA, a particular TBARS. Other groups found that LoCo post-viral chronic fatigue and affective symptoms are associated with oxidative damage, lowered antioxidant defenses, and inflammation (Al-Hakeim et al. 2023).

Several amino acids were measured by LC–MS/MS in serum samples of COVID-19 subjects and healthy controls (Atila et al. 2021). In that study, differences between COVID-19 subjects and healthy controls were found for many amino acids, notably for 2-aminobutyric acid and Phe. The authors suggested that these amino acids have biomarker potential for COVID-19, with 2-aminobutyric acid even having prognostic information about the course of the disease (Atila et al. 2021). The serum amino acid concentrations reported by Atila et al. 2021 were all in the nM-range, which is several orders of magnitude lower compared to those reported by us in this and in previous clinical studies (e.g., Hanff et al. 2017, 2019; Baskal et al. 2021a). In our study, we did not measure 2-aminobutyric acid in serum or urine samples. With respect to Phe, we did not find significant differences between LoCo and ExCo subjects.

Serum amino acids were measured by LC–MS/MS in hospitalized COVID participants and in healthy controls (Ozturk et al. 2022). This study found 27 amino acids with higher concentrations in the COVID participants, with no differences for 13 amino acids. These authors reported that alpha-aminopimelic acid, sarcosine, and hydroxyproline were considerably higher in the control group than in the COVID participant group (*P* < 0.0001) (Ozturk et al. 2022). For sarcosine (Sarc), and hydroxyproline (OH-Pro) and some other amino acids, we found differences between the LoCo and ExCo groups. However, we measured in our study normal Sarc concentrations in serum of the order of 1 µM, which is about 400 times lower than the values reported by Ozturk et al. 2022.

Metabolomics analysis by GC–MS revealed a modified amino acid metabolism that correlated with altered oxygen homeostasis in COVID-19 participants (Páez-Franco et al. 2021). α-Hydroxyl acids of amino acid origin increased with disease severity and correlated with altered oxygen saturation levels and clinical markers of lung damage. These authors concluded that amino acids might have implications on the appearance of adverse effects due to SARS-CoV-2 infection, such as diabetes and neurological disabilities (Páez-Franco et al. 2021). 4-OH-Pro plays important roles in health and nutrition (Wu et al. 2019; Wu 2020). 4-OH-Pro was found to display a trend towards higher levels in severely diseased COVID-19 participants (Páez-Franco et al. 2021). Our results indicating gender-related differences between the LoCo and ExCo with respect to 4-OH-Pro are supportive of a role of 4-OH-Pro in COVID-19. The potential importance of amino acid sensing pathways in the pathogenesis of obesity and COVID-19 has been recently discussed (Philips and Khan 2021).

Given the particular importance of Arg-involving pathways, notably including the Arg/NO pathway that generates NO, which is an endogenous multifunctional signaling molecule, we investigated this pathway in our LoCo and ExCo groups. We measured nitrite and nitrate as measures of NO synthesis, as well as Arg and ADMA, the substrate and the inhibitor of NO synthases, respectively. With respect to Arg and ADMA, we did not find appreciable differences between the LoCo and ExCo groups. This suggests that the capacity of the body to produce NO from Arg is not affected by COVID-19. The circulating and urinary concentrations of nitrate did not differ between the groups, suggesting that both systemic and whole body NO synthesis are closely comparable in the LoCo and ExCo subjects (Tsikas et al. 2006). LoCo participants in our study showed lower (by 23%) serum nitrite levels than the ExCo subjects, possibly suggesting (Kleinbongard et al. 2003, 2006) lower NO synthesis in endothelial cells and a risk of endothelial dysfunction in the LoCo participants. In this context, it is worth mentioning that nitrite may be bioactivated by its reduction to NO independent of NO synthase, thus contributing to hypoxic vasodilation, physiological blood pressure control, and redox signaling (Kim-Shapiro and Gladwin 2014). Possible mechanisms may involve reduction of nitrite to NO by hemoglobin species, xanthine oxidoreductase and carbonic anhydrase (Zinke et al. 2016), notably under hypoxic and acidic conditions (Kim-Shapiro and Gladwin 2014). The lower serum nitrite concentrations we measured in LoCo compared to ExCo may suggest that the above mentioned nitrite-reducing mechanisms were extenuated in the LoCo subjects. The serum Sarc concentrations behaved similarly to the serum nitrite concentrations: they were lower in the LoCo compared to the ExCo subjects. We are not aware of a biochemical relationship between Sarc and nitrite, with the exception that nitrite may react under acidic conditions with creatine to form the weakly carcinogenic *N*-nitroso-sarcosine (Archer et al. 1971).

The nitrate–nitrite–nitric oxide pathway has been shown to be suitable as a therapeutic means in pulmonary arterial hypertension (Sparacino-Watkins et al. 2012). The principal therapeutic use of inhaled NO in a participant with vasoreactive idiopathic pulmonary arterial hypertension and COVID-19 infection has been reported (Zamanian et al. 2020; Alvarez et al. 2020).

It has been reported that treatment of COVID-19 participants with L-arginine (1.66 g) and liposomal vitamin C (500 mg) for 28 days (Tosato et al. 2022) or 30 days (Izzo et al. 2022) improved endothelial function. This combination has been reported to improve LoCo symptoms (Izzo et al. 2022) and to increase walking capacity and muscle strength (Tosato et al. 2022). Yet, these studies did not provide mechanistic insides.

Few studies reported on the sum of nitrite and nitrate and oxidative stress in hospitalized participants with severe acute COVID-19 (Munguia et al. 2022; Orea-Tejada et al. 2022). The results of these studies with respect to nitrite and nitrate are not in line with our results.

A potential limitation of our study might be the absence of a healthy control group that was not affected at all by COVID-19 and our sampling method (convenience sampling). However, the primary aim of our study was to study potential differences between LoCo and ExCo subjects. ExCo might even represent a better control group concerning the development of LoCo symptoms after an infection by COVID-19. Both groups had low hospitalization rates, so an overestimation of possible effects of COVID-19 were not biased by intensity care. An advantage of our study is that we measured amino acids and their metabolites both in serum and in urine. This provides additional formation about the renal handling of these endogenous substances in LoCo and ExCo. As far as we are informed, our study is the first one to have investigated amino acids in LoCo participants. In contrast to our study, other groups investigated subjects in an acute phase of COVID-19. Divergences in the outcome of reported studies might be in part due to differences in study design and severity of the infected participants, and in part for analytical reasons. The lower serum nitrite concentrations we measured in our LoCo participants and the beneficial effects of inhaled NO as well as of orally administered Arg in combination with vitamin C reported by other groups, suggest that dietary or supplementary inorganic nitrite may also be beneficial in COVID-19. The analysis of amino acids and their metabolites in a bigger LoCo sample could contribute to a better understanding of the pathomechanisms of the development of long COVID.

### Supplementary Information

Below is the link to the electronic supplementary material.

Supplementary file1 (DOCX 204 KB)

## Data Availability

Data are freely available to any researcher wishing to use them for non-commercial purposes.

## Abbreviations

ADMA: Asymmetric dimethylarginine
AGAT: Arginine glycine amidinotransferase
AGEs: Advanced glycation end-products
AUC: Area under the curve
COVID-19: Coronavirus disease 2019
DBP: Diastolic blood pressure
eGFR: Estimated glomerular filtration rate
ExCo: Ex COVID-19
FE: Fractional excretion
GAA: Guanidino acetate
GAMT: Guanidinoacetate methyltransferase
GC–MS: Gas chromatography-mass spectrometry
GNMT: Glycine-*N*-methyltransferase
hArg: Homoarginine
IQR: Interquartile range
LMMA: Monomethylarginine
LoCo: Long COVID-19
MDA: Malondialdehyde
NO: Nitric oxide
NOS: NO synthase
PTM: Post-translational modification(s)
QC: Quality control
RAGE: Receptor of AGEs
ROC: Receiver operating characteristic
Sarc: Sarcosine
SARS-CoV-2: Severe acute respiratory syndrome coronavirus 2
SBP: Systolic blood pressure
sCRP: Serum C-reactive protein
SDMA: Symmetric dimethylarginine
