# Supplementary material for: Amino acids, post-translational modifications, nitric oxide, and oxidative stress in serum and urine of long COVID and ex COVID human subjects
Source: Amino Acids. 2023 Jul 29;55(9):1173–88. doi: 10.1007/s00726-023-03305-1 (PMC10564820; doi:10.1007/s00726-023-03305-1)
Supplement: Supplementary file 1 — Supplementary file1 (DOCX 204 KB) [file 726_2023_3305_MOESM1_ESM.docx]

**Supplement to**

**Amino acids, post-translational modifications, nitric oxide, and oxidative stress in serum and urine of long-COVID and ex-COVID human subjects**

Marie Mikuteit^1,2, a^, Svetlana Baskal^3,a^, Sandra Klawitter^4^, Alexandra Dopfer-Jablonka^1^, Georg M.N. Behrens^1,5,6^, Frank Müller^7,8^, Dominik Schröder^7^, Frank Klawonn^4,9^, Sandra Steffens^1,2^, Dimitrios Tsikas^3,^*

**Table S1.** Results of the GC-MS analyses of the quality control (QC) samples in serum and urine in the study. Concentrations are reported in µM for all analytes. SD, standard deviation; RSD, relative standard deviation

| Analyte | Serum (#140, *n*=5) | | | Urine (#2, *n*=5) | | |  |
| --- | --- | --- | --- | --- | --- | --- | --- |
|  | mean | SD | RSD (%) | mean | SD | RSD (%) |  |
| *Amino Acids and PTMs* | | | | | | |  |
| Ala | 223 | 30.9 | 13.9 | 115 | 2.9 | 2.5 |  |
| Asp+Asn | 53.8 | 7.2 | 13.3 | 65.6 | 1.0 | 1.5 |  |
| Glu+Gln | 533 | 68 | 12.7 | 411 | 16.8 | 4.1 |  |
| Phe | 61.6 | 8.0 | 12.9 | 20.1 | 1.1 | 5.7 |  |
| Gly | 144 | 18.3 | 12.7 | 1440 | 41 | 2.9 |  |
| Leu+Ile | 172 | 14 | 14.2 | 35.0 | 1.7 | 4.9 |  |
| Lys | 110 | 14 | 12.5 | 28.1 | 0.65 | 2.3 |  |
| Met | 52.8 | 5.0 | 9.6 | 38.6 | 0.78 | 2.0 |  |
| Pro | 111 | 13.6 | 12.3 | 6.41 | 0.20 | 3.1 |  |
| Arg | 71.9 | 9.1 | 12.6 | 6.90 | 0.31 | 4.5 |  |
| Ser | 98 | 9.5 | 9.7 | 187 | 3.3 | 1.8 |  |
| Thr | 120 | 16 | 13.7 | 71.4 | 2.7 | 3.8 |  |
| Val | 240 | 30 | 12.7 | 19.7 | 0.59 | 3.0 |  |
| Tyr | 46.7 | 5.3 | 11.4 | 34 | 0.8 | 2.4 |  |
| Sarc | 0.88 | 0.09 | 10.5 | 2.52 | 0.13 | 5.1 |  |
| Orn+Cit | 45.3 | 4.8 | 10.5 | 9.08 | 0.36 | 4.9 |  |
| GAA | 1.16 | 0.10 | 9.1 | 56.4 | 1.9 | 3.1 |  |
| hArg | 0.73 | 0.09 | 12.0 | 0.45 | 0.01 | 2.6 |  |
| OH-Pro | 4.0 | 0.4 | 10.2 | 3.37 | 0.14 | 4.2 |  |
| D-5OH-Lys | 0.27 | 0.01 | 5.0 | 0.37 | 0.08 | 2.3 |  |
| L-5OH-Lys | 0.69 | 0.02 | 3.2 | 1.42 | 0.03 | 2.1 |  |
| MML | 6.56 | 0.69 | 10.5 | 2.81 | 0.11 | 3.8 |  |
|  | | | | | | |  |
| *Creatinine, Nitrate, Nitrite, MDA* | | | | | | | Recovery (%) Serum; Urine |
| Crea-QC1 | 104 | 2.2 | 2.2 | 184 | 8 | 0.4 | not applicable |
| Crea-QC2 | 161 | 4.1 | 2.5 | 395 | 1 | 0.2 | 95 ; 106 |
| Crea-QC3 | 229 | 1.9 | 0.8 | 958 | 25 | 1.9 | 104 ; 97 |
| Nitrate-QC1 | 56.5 | 4.4 | 7.8 | 276 | 3.6 | 1.3 | not applicable |
| Nitrate-QC2 | 86.7 | 5.6 | 6.5 | 500 | 7.8 | 1.6 | 101 ; 90 |
| Nitrate-QC3 | 102 | 18 | 17.8 | 742 | 3.8 | 0.5 | 91 ; 93 |
| Nitrite-QC1 | 1.44 | 0.05 | 3.2 | 2.11 | 0.24 | 11.4 | not applicable |
| Nitrite-QC2 | 3.06 | 0.02 | 0.7 | 7.0 | 0.77 | 11.0 | 108 ; 98 |
| Nitrite-QC3 | 4.13 | 0.62 | 15.0 | 10.5 | 0.31 | 3.0 | 90 ; 84 |
| MDA-QC1 | 2.59 | 0.006 | 0.2 | 2.45 | 0.29 | 11.7 | not applicable |
| MDA-QC2 | 4.84 | 0.31 | 6.4 | 4.28 | 0.28 | 6.4 | 113; 92 |
| MDA-QC3 | 9.16 | 0.07 | 0.74 | 10.6 | 0.35 | 3.3 | 110; 102 |

**Table S2**. Serum concentrations (in µM) in the female (*n*=112) and male (*n*=36) patients of the whole study

| Analyte | females | | males | | *P*  (M-W) | *P adj.* | AUC | | *P*  (ROC) | *P adj*. (ROC) |
| --- | --- | --- | --- | --- | --- | --- | --- | --- | --- | --- |
|  | mean | SD | mean | SD |  |  | Area | SE |  |  |
| *Amino Acids and PTMs* | | | | | | | | | | |
| Ala | 341 | 93.5 | 354 | 85.2 | 0.1863 | 1.0 | 0.574 | 0.0549 | 0.1852 | 1.0 |
| Asp+Asn | 83.5 | 20.4 | 85.8 | 19.8 | 0.4755 | 1.0 | 0.540 | 0.0521 | 0.4732 | 1.0 |
| Glu+Gln | 720 | 159.6 | 776 | 154.4 | 0.0676* | 1.0 | 0.612 | 0.0512 | 0.04436 | 0.71485 |
| Phe | 68.5 | 16.2 | 70.5 | 13.0 | 0.5041* | 1.0 | 0.555 | 0.0515 | 0.3233 | 1.0 |
| Gly | 230 | 64.5 | 216 | 56.1 | 0.2299 | 1.0 | 0.567 | 0.0525 | 0.2285 | 1.0 |
| Leu+Ile | 188.2 | 54.1 | 228 | 55.0 | 0.0001 | 0.0026 | 0.709 | 0.0467 | 0.000170 | 0.00425 |
| Lys | 158.3 | 48.2 | 180.3 | 41.3 | 0.0150* | 0.3000 | 0.648 | 0.0488 | 0.007500 | 0.165 |
| Met | 63.1 | 9.77 | 66.4 | 8.83 | 0.0713* | 1.0 | 0.613 | 0.0503 | 0.04205 | 0.71485 |
| Pro | 155.6 | 60.1 | 176.3 | 58.6 | 0.0245 | 0.4410 | 0.625 | 0.0527 | 0.02490 | 0.4482 |
| Arg | 91.6 | 23.6 | 86.9 | 32.3 | 0.0901 | 1.0 | 0.594 | 0.0536 | 0.08993 | 1.0 |
| Ser | 139.0 | 30.2 | 136.6 | 26.2 | 0.9300 | 1.0 | 0.505 | 0.0546 | 0.9288 | 1.0 |
| Thr | 137.7 | 39.9 | 138.9 | 33.4 | 0.8770* | 1.0 | 0.513 | 0.0519 | 0.8214 | 1.0 |
| Val | 266 | 79.1 | 305 | 62.2 | 0.0017 | 0.0391 | 0.672 | 0.0470 | 0.00192 | 0.04416 |
| Tyr | 48.0 | 16.0 | 48.2 | 11.8 | 0.6064 | 1.0 | 0.529 | 0.0516 | 0.6042 | 1.0 |
| Sarc | 0.915 | 0.395 | 0.985 | 0.315 | 0.0757 | 1.0 | 0.599 | 0.0553 | 0.07571 | 1.0 |
| Orn+Cit | 88.7 | 29.8 | 107.9 | 32.5 | 0.0010 | 0.0240 | 0.681 | 0.0518 | 0.001111 | 0.026664 |
| GAA | 1.93 | 0.416 | 2.12 | 0.540 | 0.0344* | 0.5848 | 0.606 | 0.0571 | 0.05525 | 0.82875 |
| hArg | 1.34 | 0.553 | 1.64 | 0.628 | 0.0072 | 0.1584 | 0.648 | 0.0530 | 0.007550 | 0.165 |
| OH-Pro | 6.34 | 3.18 | 7.71 | 3.77 | 0.0135 | 0.2835 | 0.637 | 0.0534 | 0.01391 | 0.2782 |
| D-5OH-Lys | 0.321 | 0.054 | 0.327 | 0.053 | 0.5265* | 1.0 | 0.530 | 0.0537 | 0.5841 | 1.0 |
| L-5OH-Lys | 0.857 | 0.171 | 0.841 | 0.161 | 0.5337 | 1.0 | 0.535 | 0.0545 | 0.5315 | 1.0 |
| MML | 5.17 | 4.16 | 3.93 | 3.08 | 0.1863 | 1.0 | 0.574 | 0.0512 | 0.1852 | 1.0 |
| *Creatinine, Nitrate, Nitrite, MDA* | | | | | | | | | |  |
| Creatinine | 99.9 | 15.2 | 119.3 | 12.8 | < 0.0001* | 0.0026 | 0.844 | 0.0343 | < 0.0001 | 0.0026 |
| Nitrate | 67.7 | 21.4 | 63.6 | 18.2 | 0.2459 | 1.0 | 0.565 | 0.0571 | 0.2444 | 1.0 |
| Nitrite | 2.05 | 1.02 | 2.08 | 0.798 | 0.2205 | 1.0 | 0.568 | 0.0511 | 0.2191 | 1.0 |
| MDA | 1.08 | 0.384 | 1.15 | 0.244 | 0.0154 | 0.300 | 0.634 | 0.0486 | 0.01584 | 0.30096 |

***** unpaired *t* test. SD, standard deviation; SE, standard error; AUC, area under the curve; ROC, receiver operating characteristic; M-W, Mann-Whitney U test; *P* values adjusted with Bonferroni-Holm method.

**Table S3**. **Urinary creatinine concentration (in mM) and creatinine-corrected excretion rates (µM/mM) of the measured analytes in the female (*n*=112) and male (*n*=36) patients of the whole study**

| Analyte | females | | males | | *P*  (M-W) | *P adj.* | AUC | | *P*  (ROC) | *P adj. (ROC)* |
| --- | --- | --- | --- | --- | --- | --- | --- | --- | --- | --- |
|  | mean | SD | mean | SD |  |  | Area | SE |  |  |
| *Amino Acids and PTMs* | | | | | | | | | | |
| Ala | 15.5 | 9.31 | 13.1 | 5.91 | 0.3894 | 1.0 | 0.548 | 0.0562 | 0.3872 | 1.0 |
| Asp+Asn | 12.6 | 5.28 | 10.2 | 3.59 | 0.0304 | 0.6992 | 0.620 | 0.0533 | 0.0308 | 0.7073 |
| Glu+Gln | 68.0 | 25.2 | 60.5 | 21.6 | 0.1970 | 1.0 | 0.572 | 0.0556 | 0.1958 | 1.0 |
| Phe | 3.55 | 1.31 | 3.04 | 1.15 | 0.0967 | 1.0 | 0.592 | 0.0531 | 0.0965 | 1.0 |
| Gly | 100.2 | 80.4 | 64.6 | 34.7 | 0.0055 | 0.1595 | 0.653 | 0.0518 | 0.0059 | 0.1707 |
| Leu+Ile | 10.8 | 11.9 | 8.80 | 6.53 | 0.2450 | 1.0 | 0.565 | 0.0558 | 0.2435 | 1.0 |
| Lys | 8.40 | 9.54 | 8.07 | 8.52 | 0.9106 | 1.0 | 0.506 | 0.0591 | 0.9093 | 1.0 |
| Met | 5.92 | 1.88 | 4.95 | 1.66 | 0.0084 | 0.2352 | 0.646 | 0.0536 | 0.0088 | 0.2461 |
| Pro | 1.22 | 0.423 | 1.45 | 2.72 | 0.0245 | 0.5880 | 0.625 | 0.0533 | 0.0249 | 0.5981 |
| Arg | 1.52 | 0.599 | 1.38 | 0.516 | 0.3489 | 1.0 | 0.552 | 0.0553 | 0.3469 | 1.0 |
| Ser | 24.4 | 10.8 | 20.1 | 6.89 | 0.0906 | 1.0 | 0.594 | 0.0536 | 0.0904 | 1.0 |
| Thr | 15.1 | 8.32 | 12.1 | 4.98 | 0.0717 | 1.0 | 0.600 | 0.0526 | 0.0717 | 1.0 |
| Val | 3.76 | 1.29 | 3.28 | 1.08 | 0.0648 | 1.0 | 0.603 | 0.0516 | 0.0648 | 1.0 |
| Tyr | 9.23 | 3.13 | 8.32 | 3.09 | 0.2097 | 1.0 | 0.570 | 0.0557 | 0.2084 | 1.0 |
| Sarc | 0.237 | 0.119 | 0.217 | 0.149 | 0.2209 | 1.0 | 0.568 | 0.0541 | 0.2199 | 1.0 |
| Orn+Cit | 2.66 | 0.858 | 2.46 | 0.821 | 0.5471 | 1.0 | 0.534 | 0.0541 | 0.5448 | 1.0 |
| GAA | 18.8 | 10.9 | 11.4 | 5.45 | 0.0001 | 0.0032 | 0.712 | 0.0448 | 0.0001 | 0.0044 |
| hArg | 0.157 | 0.237 | 0.135 | 0.235 | 0.4118 | 1.0 | 0.546 | 0.0541 | 0.4096 | 1.0 |
| OH-Pro | 0.299 | 0.303 | 0.252 | 0.199 | 0.1539 | 1.0 | 0.579 | 0.0563 | 0.1534 | 1.0 |
| D-5OH-Lys | 0.0888 | 0.0530 | 0.078 | 0.0356 | 0.6667 | 1.0 | 0.524 | 0.0552 | 0.6647 | 1.0 |
| L-5OH-Lys | 0.394 | 0.188 | 0.320 | 0.103 | 0.0152 | 0.38 | 0.634 | 0.0520 | 0.0157 | 0.3935 |
| MML | 0.693 | 0.753 | 0.683 | 0.874 | 0.1081 | 1.0 | 0.589 | 0.0617 | 0.1077 | 1.0 |
| ADMA | 2.31 | 0.673 | 1.92 | 0.532 | 0.0093 | 0.2511 | 0.644 | 0.0485 | 0.0098 | 0.2633 |
| CML | 0.589 | 0.285 | 0.535 | 0.325 | 0.0571 | 1.0 | 0.606 | 0.0557 | 0.0573 | 1.0 |
| CEL | 0.475 | 0.220 | 0.429 | 0.190 | 0.3627 | 1.0 | 0.551 | 0.0539 | 0.3608 | 1.0 |
| CEA | 0.720 | 1.00 | 0.857 | 1.34 | 0.7444 | 1.0 | 0.518 | 0.0586 | 0.7426 | 1.0 |
| CEC | 0.585 | 0.451 | 0.544 | 0.558 | 0.5771 | 1.0 | 0.531 | 0.0553 | 0.5749 | 1.0 |
| Furosine | 0.075 | 0.0503 | 0.0554 | 0.0452 | 0.0015 | 0.045 | 0.674 | 0.0522 | 0.0017 | 0.0506 |
| Hypusine | 0.181 | 0.0488 | 0.176 | 0.0585 | 0.8751 | 1.0 | 0.509 | 0.0587 | 0.8739 | 1.0 |
| *Creatinine, Nitrate, Nitrite* | | | | | | | | | | |
| Creatinine | 9.92 | 9.04 | 13.0 | 10.4 | 0.0453 | 0.9966 | 0.611 | 0.0515 | 0.0456 | 1.0 |
| Nitrate | 85.1 | 62.4 | 58.9 | 27.8 | 0.0012 | 0.0372 | 0.678 | 0.0526 | 0.0013 | 0.0412 |
| Nitrite | 0.392 | 0.587 | 0.219 | 0.137 | 0.0104 | 0.2704 | 0.641 | 0.0496 | 0.0109 | 0.2829 |

SD, standard deviation; SE, standard error; AUC, area under the curve; ROC, receiver operating characteristic; M-W, Mann-Whitney U test; *P* values adjusted with Bonferroni-Holm method.

**Table S4. Fractional Excretion values of measured analytes in all groups.**

Fractional excretion (FE, %) values (mean with standard deviation, SD) were calculated for all analytes by dividing the concentration ratio of creatinine in serum and urine by the concentration ratio of an analyte in serum and urine, and by multiplying the outcome by 100.

| **NO3** | **NO2** | Ala | Thr | Gly | Val | Ser | Sarc Leu | **GAA** | Asp | OHPro | Pro | Met | Glu | OHLD | OHKL | Orn | Phe | Tyr | Lys |  | Arg | MML | hArg |
| --- | --- | --- | --- | --- | --- | --- | --- | --- | --- | --- | --- | --- | --- | --- | --- | --- | --- | --- | --- | --- | --- | --- | --- |

| **All** |  |  | **11,9** | **2,06** | 0,455 | 1,09 | 4,14 | 0,145 | 1,78 | 2,81 | 0,557 | 90,4 | 1,54 | 0,475 | 0,0874 | 0,944 | 0,982 | 2,79 | 4,65 | 0,313 | 0,535 | 2,11 | 0,578 | 0,180 | 1,81 | 1,22 |
| --- | --- | --- | --- | --- | --- | --- | --- | --- | --- | --- | --- | --- | --- | --- | --- | --- | --- | --- | --- | --- | --- | --- | --- | --- | --- | --- |
| **SD** |  |  | **6,05** | **3,47** | 0,266 | 0,536 | 2,77 | 0,064 | 0,820 | 1,63 | 0,543 | 56,1 | 0,727 | 0,445 | 0,072 | 0,390 | 0,498 | 1,43 | 1,90 | 0,131 | 0,247 | 1,05 | 0,791 | 0,085 | 2,61 | 2,08 |
|  |  |  |  |  |  |  |  |  |  |  |  |  |  |  |  |  |  |  |  |  |  |  |  |  |  |  |
| **LoCo** |  |  | **11,9** | **2,19** | 0,452 | 1,10 | 4,04 | 0,148 | 1,76 | 2,79 | 0,540 | 94,9 | 1,53 | 0,463 | 0,0827 | 0,948 | 0,981 | 2,69 | 4,66 | 0,315 | 0,537 | 2,15 | 0,611 | 0,182 | 1,89 | 1,31 |
| **SD** |  |  | **6,36** | **3,75** | 0,283 | 0,571 | 2,73 | 0,1 | 0,9 | 1,45 | 0,503 | 58,1 | 0,738 | 0,409 | 0,038 | 0,412 | 0,529 | 1,42 | 1,99 | 0,136 | 0,260 | 1,10 | 0,86 | 0,089 | 2,82 | 2,24 |
|  |  |  |  |  |  |  |  |  |  |  |  |  |  |  |  |  |  |  |  |  |  |  |  |  |  |  |
| **ExCo** |  |  | **11,7** | **1,36** | 0,471 | 1,06 | 4,70 | 0,129 | 1,94 | 2,90 | 0,645 | 67,4 | 1,57 | 0,542 | 0,112 | 0,923 | 0,986 | 3,30 | 4,61 | 0,301 | 0,525 | 1,93 | 0,404 | 0,169 | 1,37 | 0,715 |
| **SD** |  |  | **4,14** | **1,07** | 0,148 | 0,293 | 2,92 | 0,036 | 0,595 | 2,37 | 0,709 | 36,2 | 0,668 | 0,596 | 0,154 | 0,251 | 0,285 | 1,39 | 1,30 | 0,100 | 0,161 | 0,658 | 0,183 | 0,060 | 0,966 | 0,729 |
|  |  |  |  |  |  |  |  |  |  |  |  |  |  |  |  |  |  |  |  |  |  |  |  |  |  |  |
| **Female** | |  | **12,2** | **2,26** | 0,456 | 1,11 | 4,31 | 0,148 | 1,78 | 2,78 | 0,584 | 98,8 | 1,56 | 0,483 | 0,0841 | 0,951 | 0,983 | 2,75 | 4,67 | 0,317 | 0,537 | 2,07 | 0,565 | 0,173 | 1,74 | 1,24 |
| **SD** |  |  | **6,66** | **3,93** | 0,277 | 0,557 | 2,94 | 0,063 | 0,839 | 1,40 | 0,591 | 60,1 | 0,749 | 0,423 | 0,0323 | 0,369 | 0,501 | 1,42 | 1,99 | 0,122 | 0,250 | 0,970 | 0,751 | 0,084 | 2,35 | 2,00 |
|  |  |  |  |  |  |  |  |  |  |  |  |  |  |  |  |  |  |  |  |  |  |  |  |  |  |  |
| **Male** |  |  | **10,9** | **1,43** | 0,454 | 1,05 | 3,63 | 0,135 | 1,81 | 2,90 | 0,472 | 64,6 | 1,47 | 0,453 | 0,098 | 0,921 | 0,980 | 2,91 | 4,60 | 0,300 | 0,528 | 2,23 | 0,618 | 0,202 | 2,03 | 1,13 |
| **SD** |  |  | **3,40** | **1,01** | 0,231 | 0,465 | 2,06 | 0,064 | 0,758 | 2,20 | 0,345 | 28,3 | 0,652 | 0,509 | 0,134 | 0,450 | 0,488 | 1,44 | 1,57 | 0,154 | 0,237 | 1,25 | 0,903 | 0,086 | 3,28 | 2,33 |
|  |  |  |  |  |  |  |  |  |  |  |  |  |  |  |  |  |  |  |  |  |  |  |  |  |  |  |
| **LoCo female** | |  | **12,2** | **2,36** | 0,453 | 1,10 | 4,21 | 0,149 | 1,74 | 2,77 | 0,555 | 101,6 | 1,53 | 0,481 | 0,083 | 0,946 | 0,972 | 2,68 | 4,66 | 0,315 | 0,533 | 2,07 | 0,582 | 0,175 | 1,78 | 1,28 |
| **SD** | |  | **6,88** | **4,14** | 0,287 | 0,580 | 2,89 | 0,066 | 0,851 | 1,42 | 0,530 | 61,5 | 0,733 | 0,443 | 0,033 | 0,378 | 0,519 | 1,40 | 2,05 | 0,125 | 0,256 | 0,994 | 0,794 | 0,088 | 2,47 | 2,10 |
|  |  |  |  |  |  |  |  |  |  |  |  |  |  |  |  |  |  |  |  |  |  |  |  |  |  |  |
| **LoCo male** | |  | **10,9** | **1,54** | 0,447 | 1,08 | 3,36 | 0,144 | 1,81 | 2,87 | 0,480 | 68,5 | 1,54 | 0,391 | 0,081 | 0,954 | 1,02 | 2,73 | 4,66 | 0,315 | 0,553 | 2,44 | 0,727 | 0,210 | 2,34 | 1,42 |
| **SD** | |  | **3,42** | **1,10** | 0,268 | 0,537 | 1,83 | 0,074 | 0,859 | 1,54 | 0,375 | 29,9 | 0,758 | 0,215 | 0,053 | 0,524 | 0,564 | 1,50 | 1,73 | 0,172 | 0,275 | 1,42 | 1,06 | 0,091 | 3,86 | 2,74 |
|  |  |  |  |  |  |  |  |  |  |  |  |  |  |  |  |  |  |  |  |  |  |  |  |  |  |  |
| **ExCo female** | |  | **12,3** | **1,50** | 0,472 | 1,12 | 5,09 | 0,140 | 2,05 | 2,85 | 0,805 | 77,4 | 1,78 | 0,497 | 0,092 | 0,987 | 1,06 | 3,29 | 4,74 | 0,330 | 0,570 | 2,09 | 0,433 | 0,158 | 1,41 | 0,927 |
| **SD** | |  | **4,63** | **1,28** | 0,176 | 0,335 | 3,25 | 0,040 | 0,675 | 1,23 | 0,903 | 42,3 | 0,824 | 0,216 | 0,023 | 0,287 | 0,314 | 1,52 | 1,43 | 0,098 | 0,192 | 0,765 | 0,197 | 0,046 | 1,06 | 0,916 |
|  |  |  |  |  |  |  |  |  |  |  |  |  |  |  |  |  |  |  |  |  |  |  |  |  |  |  |
| **ExCo male** | |  | **11,0** | **1,19** | 0,470 | 0,995 | 4,25 | 0,115 | 1,80 | 2,96 | 0,455 | 55,63 | 1,31 | 0,595 | 0,135 | 0,846 | 0,895 | 3,32 | 4,46 | 0,267 | 0,472 | 1,74 | 0,369 | 0,183 | 1,33 | 0,463 |
| **SD** | |  | **3,35** | **0,713** | 0,106 | 0,214 | 2,40 | 0,024 | 0,448 | 3,23 | 0,261 | 21,95 | 0,229 | 0,845 | 0,224 | 0,170 | 0,214 | 1,20 | 1,10 | 0,090 | 0,087 | 0,433 | 0,158 | 0,071 | 0,846 | 0,225 |

**Figure S1.** Fractional excretion rates of (A) homoarginine (hArg) and (B) guanidino acetate (GAA) in the study groups (mean with standard deviation). Mann-Whitney test was used to test statistical difference between females and males in the whole group and in the LoCo and ExCo groups. See also Table S4.
